# Large-Scale, Bandwidth-Adjustable, Visible Absorbers by Evaporation and Annealing Process

**DOI:** 10.1186/s11671-019-2881-6

**Published:** 2019-02-06

**Authors:** Xiyu Long, Weisheng Yue, Yarong Su, Weidong Chen, Ling Li

**Affiliations:** 1 College of Physics and Electronic Engineering, Sichuan Normal University, Chengdu, 610101 China; 2 State Key Laboratory of Optical Technologies on Nano-Fabrication and Micro-Engineering, Institute of Optics and Electronics, Chinese Academy of Sciences, P.O. Box 350, Chengdu, 610209 China

**Keywords:** Metasurfaces, Visible absorbers, Bandwidth-adjustable, Copper-silver alloy

## Abstract

Optical absorbers have received a significant amount of attention due to their wide range of applications in biomedical sensing, solar cell, photon detection, and surface-enhanced Raman spectroscopy. However, most of the optical absorbers are fabricated with high-cost sophisticated nanofabrication techniques, which limit their practical applications. Here, we introduce a cost-effective method to fabricate an optical absorber by using a simple evaporation technique. The absorbers are composed of evaporated nanoparticles above a silver (Ag) mirror separated by a silicon oxide layer. Experimental results show over 77% absorption in the wavelength range from 470 to 1000 nm for the absorber with isolated Ag nanoparticles on the top. The performance of the absorber is adjustable with the morphology and composition of the top-layer nanoparticles. When the top layer was hybrid silver-copper (Ag-Cu) nanoparticles (NPs), the absorption exceeding 90% of the range of 495–562 nm (bandwidth of 67 nm) was obtained. In addition, the bandwidth for over 90% absorption of the Ag-Cu NP absorber was broadened to about 500 nm (506–1000 nm) when it annealed at certain temperatures. Our work provides a simple way to make a highly efficient absorber of a large area for the visible light, and to transit absorption from a narrow band to broadband only by temperature treatment.

## Introduction

Sub-wavelength absorbers have attracted considerable attentions due to their light and thin features which enable their wide applications ranging from biochemical sensing [1, 2], and enhanced spectroscopies to solar cells [3–5]. Classical metal-insulator-metal (MIM) absorbers consist of top-layer metallic resonators and a bottom metal mirror separated by a spacer layer. The absorption of light can be maximized when a large number of plasmonic nanostructures are exposed to incident light with suitable frequency [6, 7]. As the absorption is associated with excitation of local surface plasma resonances (LSPRs) of the patterned structures, it is possible to adjust the absorption by changing the structural design [8–10]. In addition, changing the material of the spacer layer results in the change of absorption. Some phase-change materials like Ge_2_Sb_2_Te_5_ [11–13] and VO_2_ [14, 15] and electrically tunable graphene [16–19] are typically used to adjust absorption. These ways break the limitations of the material’s inherent response spectrum [20, 21]. Due to the extremely fine features of the resonators, nanofabrication methods are commonly used to fabricate plasmonic absorbers. DUV lithography [22–24], nanoimprint lithography [25, 26], and electron beam lithography are mostly used nanofabrication techniques. Due to the flexibility of nanofabrication technique, various kinds of metallic structures such as gratings and nanoparticles have been fabricated and investigated for their absorption [27–30]. However, these nanofabrication techniques are expensive and complicated and not suitable for fabrication over large areas, hindering commercialization of optical absorbers. In addition, once the absorbers are fabricated, their absorption bandwidth is not easy to adjust. Recently, direct evaporation or sputtering of non-uniform nanoparticles have been introduced as low-cost methods for fabrication of plasmonic absorbers [31, 32]. These methods are promising to act as a low-cost fabrication method for optical absorbers and need to be further investigated. Especially, the fabrication of bandwidth-adjustable absorbers with the evaporation methods has not been reported.

In this work, we investigate the methods of evaporation to fabricate optical absorbers numerically and experimentally. Broadband and narrow-band absorbers were controlled by the composition of the evaporated metals. The nanoparticles were evaporated above the Ag mirror with a SiO_2_ spacer layer in between. Broadband absorption was obtained with Ag-only nanoparticles, and narrow-band absorption was obtained with hybrid Ag-Cu nanoparticles. The absorption can be converted from narrow- to broadband with the Ag-Cu nanoparticle (NP) absorber by changing the annealing temperature.

## Methods

### Fabrication of Metasurfaces

**Fig. 1:**
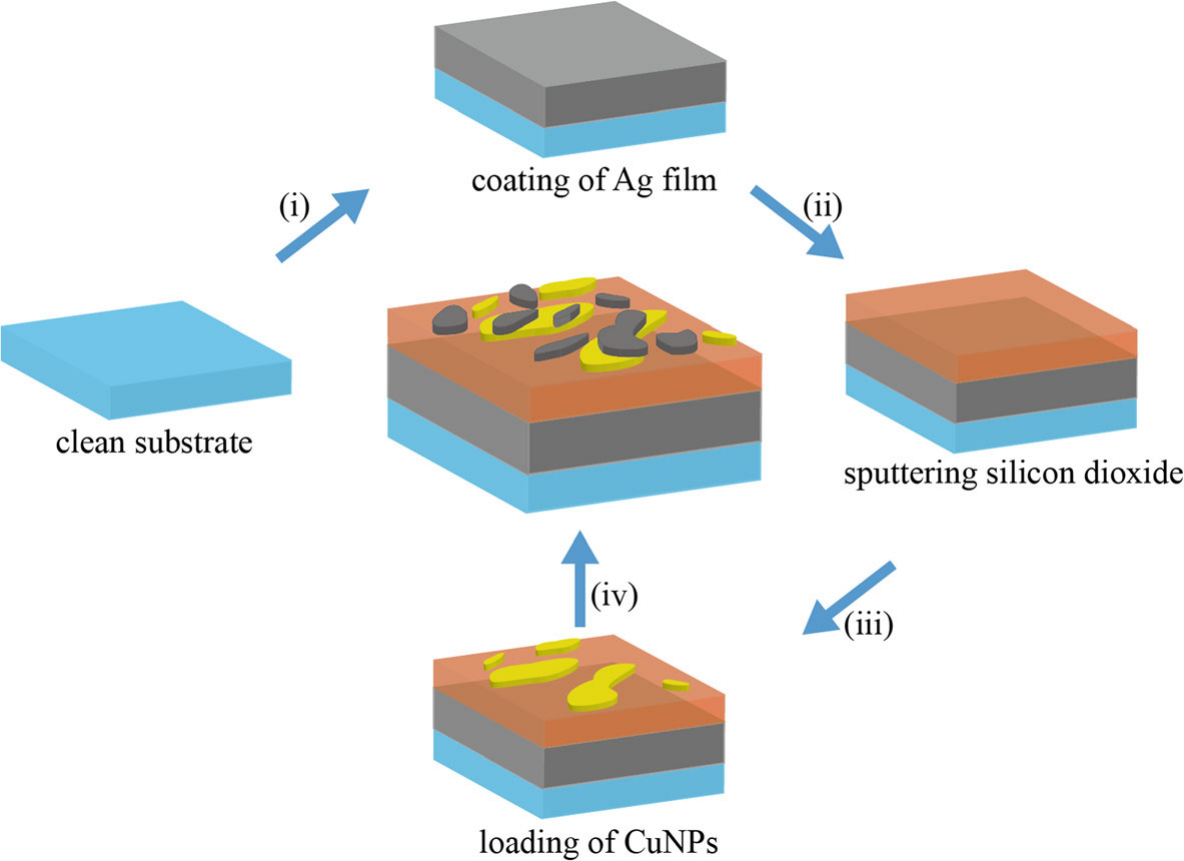
Schematic illustration of steps taken to fabricate the absorber which consists of silver and copper nanoparticles deposited on the surface: (i) coating of Ag film for counter-transmission, (ii) sputtering silicon dioxide, (iii) deposited a layer of copper particles by electron beam evaporation system, (iv) loading of Ag NPs by evaporation

The designed Ag NP and Ag-Cu NP absorbers were fabricated with evaporation methods using e-beam evaporator (DZS-500). Figure 1 shows the fabrication process: (1) 2 × 2 cm^2^ microscope glass slides were used as substrates. They were sequentially sonicated in acetone, ethanol, and deionized water for 15 min. (2) The substrates were deposited with a 15-nm-thick Ag film (deposition rate 2.5 Å/s) as a ground plane and a 90-nm SiO_2_ film (deposition rate 1 Å/s) as a spacer layer. (3) Evaporation of top-layer nanoparticles. For the Ag-Cu NP absorber, a silver nanoparticle layer was evaporated on top of a Cu nanoparticle layer to form a hybrid Ag-Cu nanoparticle absorber. The thicknesses of the Ag and Cu nanoparticle layers are both 10 nm, and the deposition rates are both 0.2 Å/s.

### Topographic Analysis

The surface patterns were examined by scanning electron microscopy (Hitachi SU8010) and atomic force microscopy (Dimension EDGE).

### Optical Analysis

The fabricated absorbers were measured with the portable spectrometer (Ocean Optics) for their reflectance. The light source is a 100-W halogen lamp. The light shines normally to the sample surface with a hybrid fiber and a holder. The measured reflection spectra were normalized to the reflection of a blank aluminum mirror.

### FEM Simulations

Numerical simulations were performed with finite-element method (FEM)-based commercial software package, CST Microwave Studio. Dispersion parameters of the Ag and Cu were obtained from literature [33]. The thickness of ground plane and dielectric layer are 150 nm and 90 nm, respectively. Unit cell boundary condition is applied in *x*- and *y*-directions. In the *z*-direction, we chose an open boundary condition. The polarization of the incident light is along the *x*-direction. As the thickness of the metallic ground plane is greater than its skin depth, the transmittance can be neglected. Then the absorption can be simplified as *A*(*ω*) = 1 − *R*(*ω*), where *R* is reflectance. To model the random distribution features of metallic nanoparticles, we changed the size and height of the particles in the simulation. The overall absorption spectrum was an enveloped profile of each individual nanoparticle simulated.

## Results and Discussions

**Fig. 2:**
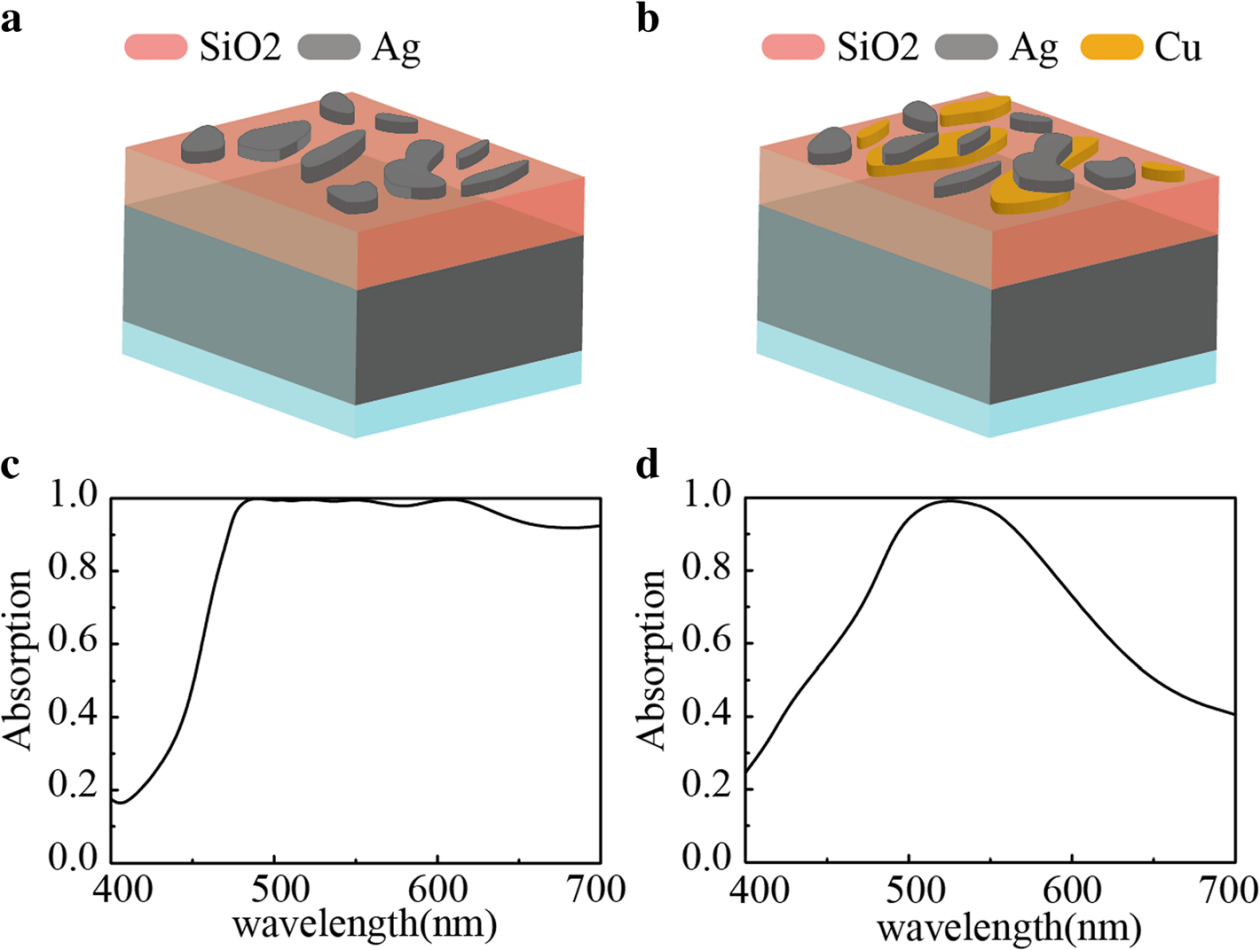
Schematics of the absorbers and simulated absorption spectra of the Ag NP and Ag-Cu NP absorbers. In these two absorbers, the carrier is glass and the underlying metal and dielectric layers are silver and silicon dioxide. **c** and **d** respectively show the absorption spectra of Ag NP absorber and Ag-Cu NP absorber structure simulation

We designed MIM absorbers with silver nanoparticles and hybrid Ag-Cu nanoparticles, respectively. The Ag NP absorber is illustrated in Fig. 2a. It consists of a continuous silver film as a ground plane, and a SiO_2_ spacer layer and Ag nanoparticles on the top as resonators. The Ag-Cu NP absorber is formed by inserting a layer of copper particles between the silver particles and the silica, as shown in Fig. 2b. Figure 2c and d show the calculated absorption spectra of the Ag NP and Ag-Cu NP absorbers, respectively. These spectrograms obtained by fitting indicate that the addition of copper does inhibit the absorption properties of the original structure.

**Fig. 3:**
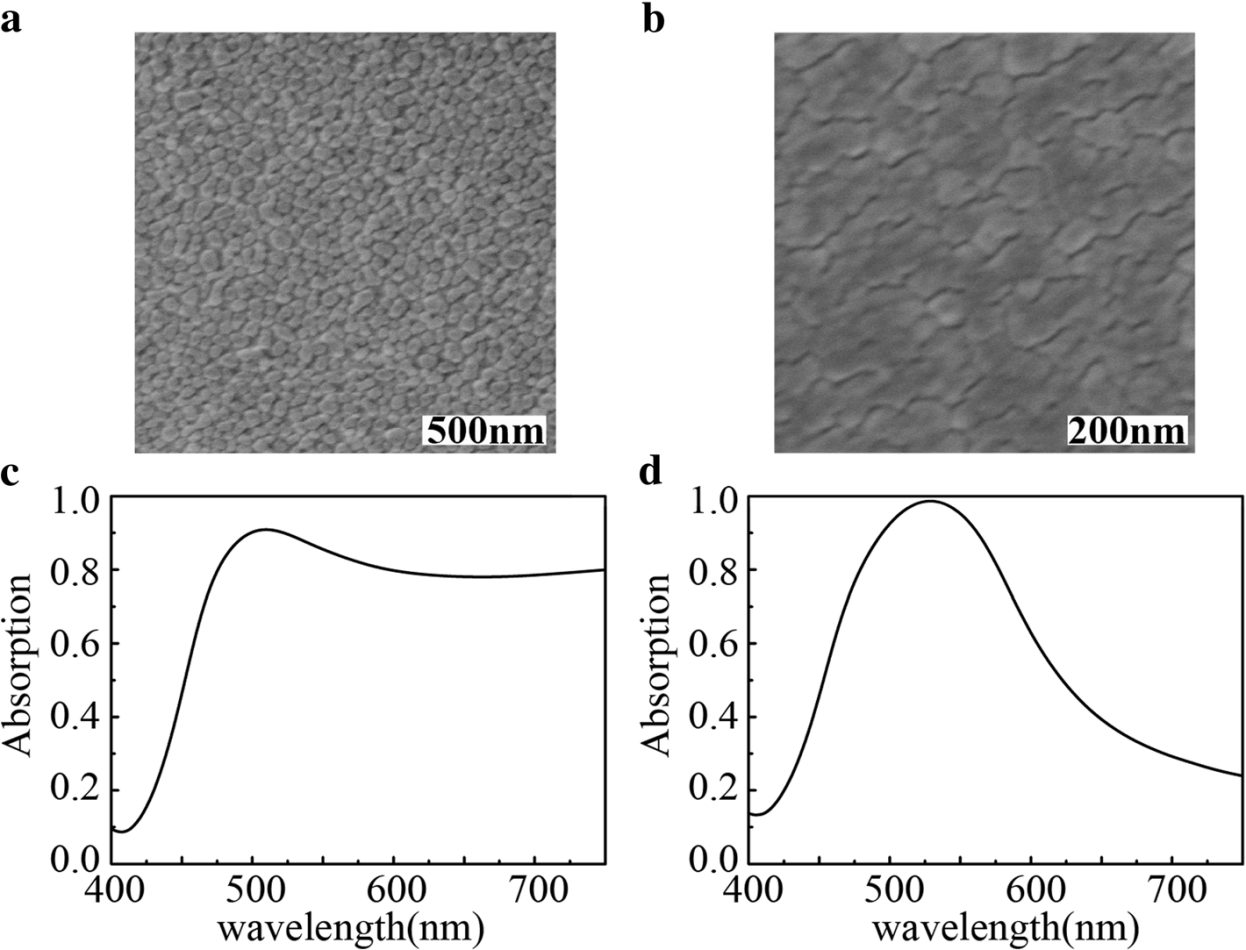
SEM image of Ag NP (**a**) and Ag-Cu NP (**b**) absorbers and corresponding and measured absorption spectrum (**c**) and (**d**)

Figure 3a and b show SEM images of the fabricated Ag NP absorber and the Ag-Cu NP absorber. From the SEM images, we can see that each nanoparticle is isolated and the boundaries are clear, indicating the successful fabrication process. Figure 3c and d present the measured absorption spectrums of the Ag NP absorber and the Ag-Cu NP absorber, respectively. The absorption of the Ag NP absorber is over 77% for the wavelength range larger than 470 nm (Fig. 3c). The absorption spectrum of the Ag-Cu NP absorber is different from that of the Ag NP absorber, as shown in Fig. 3d. The absorption bandwidth in the spectrum is much narrower in comparison to Fig. 3c. Over 80% absorption is in the range 480–577 nm with a peak of 98.6% at 528 nm leading to a narrow bandwidth of 97 nm. These results suggest that the Cu promoted the absorption of the Ag-Cu NP absorber in a narrow wavelength range while it suppressed the absorption for other wavelengths. The simulated results agree with the experimental results in the spectrum shape and resonances. The difference between the absorption intensity of the simulation with that of the experiment was caused by the difference between the actual shape of nanoparticles and the model. In the experiments, the actual shape and size of the nanoparticles were randomly distributed which were very difficult to model in the simulation. In addition, the difference of the environment between simulation and experiments also caused the difference.

**Fig. 4:**
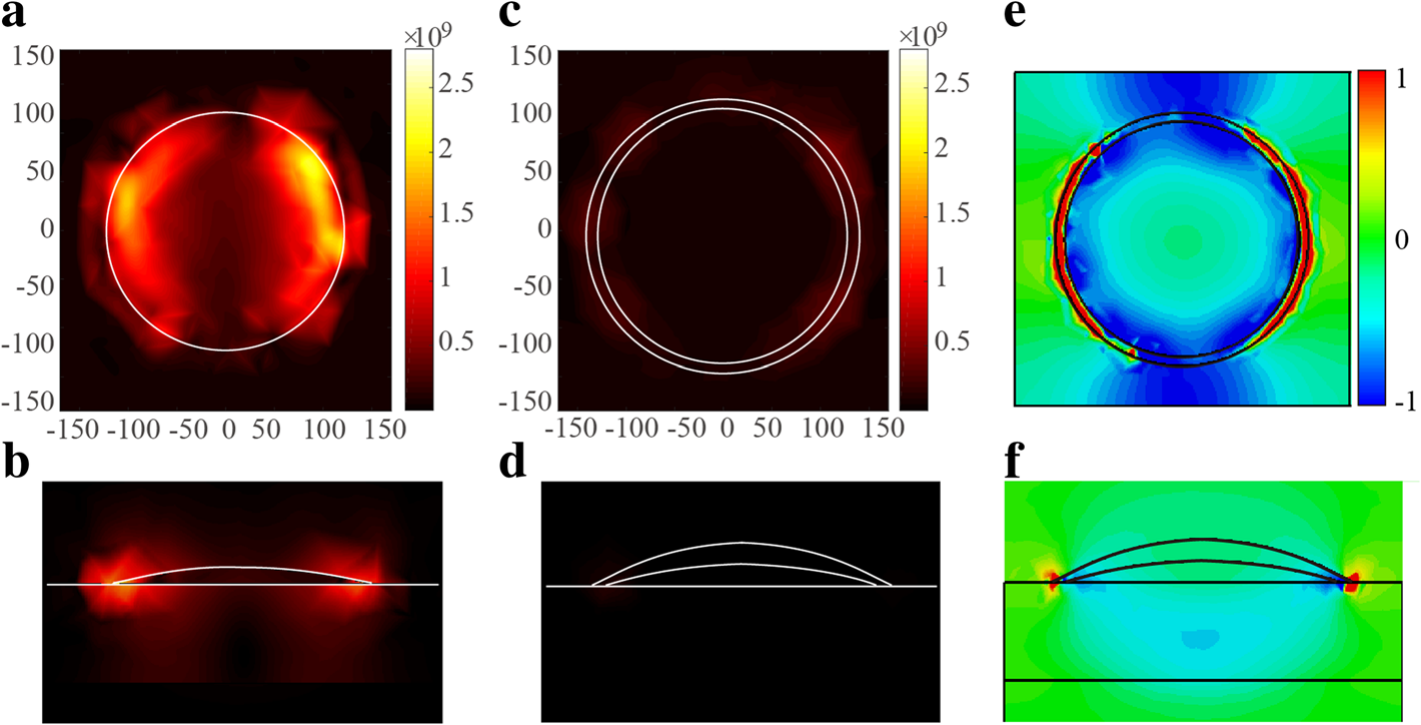
Simulated electric field distributions of **a**, **c** Ag and **b**, **d** Ag-Cu absorbers from the top and *yz* cross-section view, respectively. The Ag-Cu absorber’s *E*_*y*_ in TE mode is shown in **e** and **f**

To further understand the physics behind the observations, the electromagnetic field distribution of the absorbers was simulated. Figure 4a–d show the electric field distribution of the Ag and Ag-Cu NP absorbers, respectively. The field distributions were obtained at a resonance of 430 THz. For the Ag NP absorber, the high field intensity is at the edge of the metal particles. While for the Ag-Cu NP absorber, hot spots appear at the edge of the silver shell with intensity much lower than that of the Ag NP absorber, indicating that the Cu core has negative effects on the field enhancement of the Ag nanoparticle. A possible cause was that the Cu core reduced the interaction area of Ag particles with the bottom metal film. The field distribution of the Ag and Ag-Cu NP absorbers explained why the absorption of the Ag-Cu NP absorber was lower than that of the Ag absorber. It is noted that the Ag-Cu NP absorber has an absorption peak (> 98%) at 528 nm (see Figs. 1 and 3). In order to understand this effect, we present the field component *E*_*y*_ in Fig. 4e and f. From Fig. 4e and f, one can see that electrical dipoles within the silver shell are excited. The dipole and dipole-based resonances can lead to a high absorption when a certain wave vector component matches that of a SPP wave at the reflector-spacer interface. Experiments have also shown that the absorption peak position of the Ag-Cu NP structure can be adjusted by changing parameters such as the thickness of the dielectric layer. This property indicates that we can design resonance tunable photonic devices in a simple way.

Experiments have shown that the absorption of the Ag-Cu NPs greatly depends on the relative amount of Ag and Cu. To reveal the relationship between the thickness of these two metal layers and the absorption of Ag-Cu NP absorber, we studied the dependence of absorption on atomic number ratio *Q* of the two metals. The *Q* is defined as,

1 $$ Q=\frac{n_{\mathrm{Cu}}}{n_{\mathrm{Ag}}}=\frac{{\mathrm{Sh}}_{\mathrm{Cu}}{\rho}_{\mathrm{Cu}}}{M_{\mathrm{Cu}}}\times \frac{M_{\mathrm{Ag}}}{{\mathrm{Sh}}_{\mathrm{Ag}}{\rho}_{\mathrm{Ag}}} $$

where the density *ρ*_Ag_ is 10.53 g/cm^3^ and *ρ*_Cu_ is 8.9 g/cm^3^. Molar mass of copper (*M*_Cu_) and silver (*M*_Ag_) are 64 g/mol and 108 g/mol, respectively. The silver film was 10 nm thick, and *Q* can be changed by changing the thickness of the copper film.

**Fig. 5:**
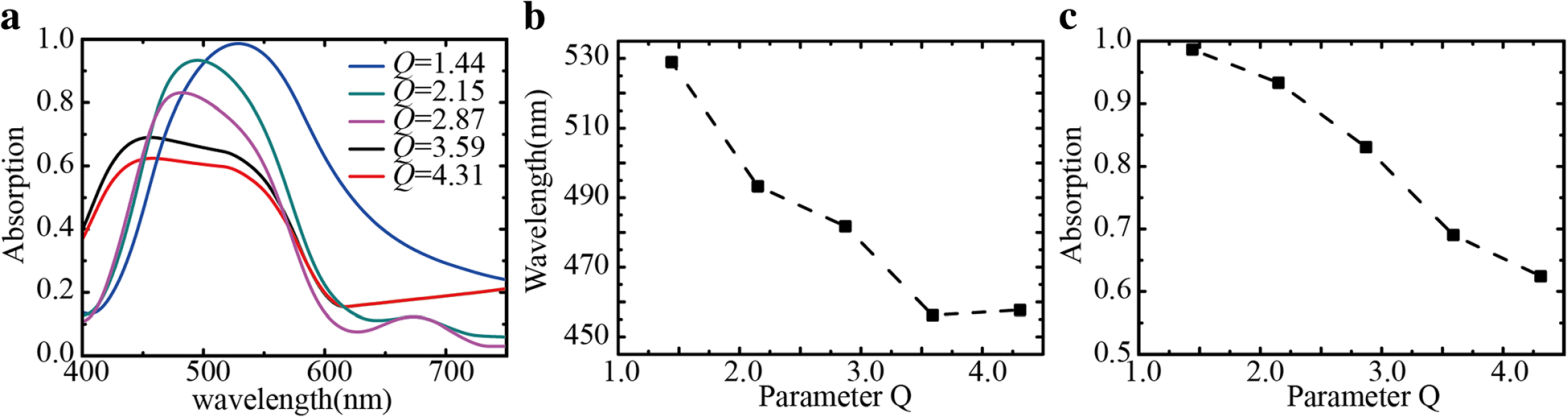
Dependence of resonance on atomic ratio *Q*. **a** Absorption spectra for different *Q* parameters. **b** Dependence of peak wavelength on *Q* and its **c** dependence of peak intensity on *Q*

Figure 5a shows absorption spectra of the Ag-Cu NP absorbers with different atomic ratio *Q*. The curves show a strong correlation between *Q* and the absorption intensity. When the *Q* increases from 1.44 to 2.15, 2.87, 3.59, and 4.31, the absorption peak shifts to lower wavelengths and the intensity decreases. Figure 5b and c are the plots of resonance peak wavelength vs. *Q* and peak intensity vs. *Q*, respectively. The two plots reveal that the resonance wavelength and the peak intensity decrease almost linearly with the increase of the atomic ratio *Q*. Previous studies have shown that the resonant wavelength is related to the size and shape of metallic nanoparticles, and the intensity is related to the surface plasmon oscillation of the metal particles [8, 34]. The change of *Q* by adjusting the thickness of the Cu film led to the absence of a continuous film and the change of the size of the particles. As the number of the gaps between nanoparticles decreases, the intensity of the optical cavities which were formed between the nanoparticles and the silver film becomes weaker. When *Q* is 1.44, the absorbance is 98.7%. When *Q* is increased to 3.59, the absorption peak position is basically stable near 460 nm. This suggests that the *Q* value is most conducive to the production of absorbers, which provides a reference for the next step and future research.

### Bandwidth Adjustment

**Fig. 6:**
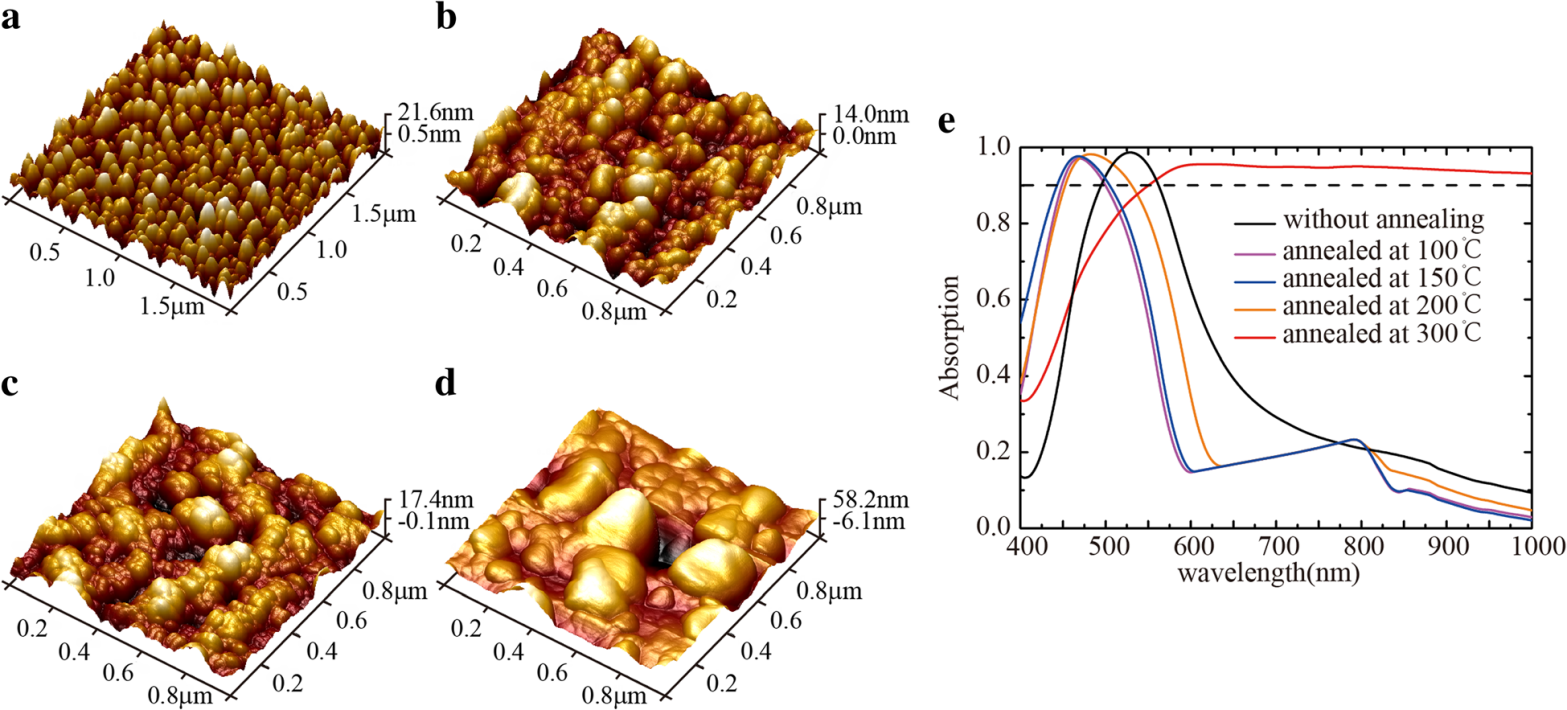
AFM images and absorption curves of Ag-Cu NP absorbers. **a** Without annealing, **b** annealed at 100 °C, **c** annealed at 150 °C, and **d** annealed at 300 °C. **e** The absorption curves of the absorber annealed at different temperatures

One of the important features of our fabricated nanoparticle absorbers is that the absorption bandwidth can be adjusted by annealing temperature. When the annealing temperatures increased from 100 to 150 °C, the absorption peak shifted to lower wavelengths. When the annealing temperatures further increased to 300 °C, the absorption peak exhibited a broadband feature. Figure 6 shows the absorption spectrum of samples which annealed at different temperatures in a vacuum annealing furnace. By raising the temperature, annealing can redistribute the metal on the surface and obtain different morphology. Surface morphology was characterized with atomic force microscopy (AFM). The AFM images shown in Fig. 6a–d are for the sample without annealing and annealing at 100 °C, 150 °C, and 300 °C, respectively. As the annealing temperature increases, the size of the metal particles and the roughness increases. When the temperature reached 100 °C, the metal particles became clustered. If the external effect is lower than the adhesion between the medium and the metal, many fine particles remain on the surface of the medium. This is the reason that the particles produced by annealing at 100 °C have smaller particle sizes. According to the absorption spectrum of Fig. 6, we can also find that annealing within a certain temperature range has little effect on the absorption performance of the Ag-Cu NP structure. However, when the temperature rises to 300 °C, its influence cannot be ignored.

**Fig. 7:**
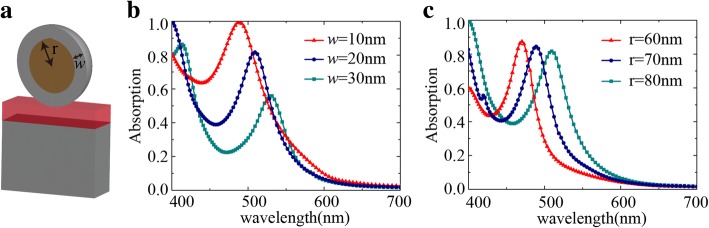
Simulation of the Ag-Cu NPs structure with Cu-Ag alloy on the surface. **a** Schematic of the model. **b** Absorption spectra with change of thickness *w*. **c** Absorption with changes of radius *r*

The absorption bandwidth extended to 494 nm (band from 506 to 1000 nm) with absorption over 90% after annealing at 300 °C. This bandwidth is significantly broad in comparison with other reported similar broadband metasurfaces. For those reported metasurfaces, the bandwidth is mostly in the range of 250~450 nm [31, 35, 36] covering only the visible range. However, our absorber is suitable for both visible and near-infrared regions with absorption intensity of 90% and above. Due to the extremely thin thickness, the melt-point temperature of the metal is much lower than that of the bulk materials. The heating causes the two metals to form nanoclusters and merge into each other at the interface due to fusing, which may result in a formation of nano-alloy with low energy and stability [37, 38]. Due to the limited amount of Ag atoms, the Ag atoms tend to converge to the surface of the cluster with Cu atoms in the center, forming a core-shell structure [39, 40]. This core-shell structure determined the features of the absorption spectra. It is known from the measured AFM image that the size of the metal particles increases with the increase of the annealing temperature. To reveal the relationship between the absorption and temperature, we calculated a core-shell model on the MIM structure. The simulated results show that increasing the Cu core’s radius and the thickness of Ag shell will lead to a shift of absorption to longer wavelengths (Fig. 7). Therefore, the red-shift and broadening of the spectrum after annealing at 300 °C was because the high temperature produced the nano-alloy and then the fine particles converge into larger-sized particles. In summary, under a certain annealing temperature, the Ag-Cu structures changed from initial selective absorption to broadband absorption. It provides a way to achieve different performance with simple operations.

## Conclusion

In conclusion, we have demonstrated fabrication of plasmonic absorbers simply with an evaporation method. Broadband and adjustable band absorbers were fabricated by controlling the composition of the evaporated nanoparticles. Broadband absorption was achieved with pure Ag nanoparticles on the top, and bandwidth-adjustable absorption was achieved with hybrid Ag-Cu nanoparticles on the top. The Ag-Cu NP absorber demonstrated single-frequency absorption before annealing and the absorption became broadband when annealed at a certain temperature. The absorption is > 90% in a wavelength range of 506–1000 nm, which covers both the visible and near-infrared ranges. Our work has provided a simple and low-cost fabrication technique to make large-area visible absorbers. In addition, the high absorption is accompanied with a huge local field enhancement, which makes our absorbers suitable for surface-enhanced Raman scattering (SERS) and other surface spectroscopies.

## Abbreviations

AFM: Atomic force microscopy
Ag: Silver
Cu: Copper
DUV: Deep ultraviolet
FEM: Finite-element method
LSPRs: Local surface plasma resonances
MIM: Metal-insulator-metal
NPs: Nanoparticles
SEM: Scanning electron microscopy
THz: Terahertz
